# Effectiveness of an educational group intervention in primary healthcare for continued exclusive breast-feeding: PROLACT study

**DOI:** 10.1186/s12884-018-1679-3

**Published:** 2018-02-26

**Authors:** Susana Martín-Iglesias, M. Jesús Santamaría-Martín, Ahinoa Alonso-Álvarez, Milagros Rico-Blázquez, Isabel del Cura-González, Ricardo Rodríguez-Barrientosn, Aurora Barberá-Martín, Teresa Sanz-Cuesta, M. Isabel Coghen-Vigueras, Isabel de Antonio-Ramírez, Isabel Durand-Rincón, Felisa Garrido-Rodriguez, María Jesús Geijo-Rincón, Rebeca Mielgo-Salvador, M. Soledad Morales-Montalvá, M. Asunción Reviriego-Gutiérrez, Carmen Rivero-Garrido, Micaela Ruiz-Calabria, M. Pilar Santamaría-Mechano, Roberto Santiago-Fernández, M. Isabel Sillero-Quintana, Beatriz Soto-Almendro, María Terol-Claramonte, María Villa-Arranz

**Affiliations:** 1 Direccion Asistencial Sur de Atencion Primaria, Servicio Madrileño de Salud, Avenida Juan de la Cierva, s/n, 28902 Getafe, Spain; 2 Centro de Salud Lucero, Servicio Madrileño de Salud, Calle Latina, 14, 28047 Madrid, Spain; 3 Unidad de Apoyo a la Investigacion Gerencia Asistencial de Atencion Primaria, Red de investigacion en Servicios de Salud en enfermedades cronicas REDISSEC, Servicio Madrileño de Salud, Calle San Martín de Porres, 6, 28035 Madrid, Spain; 4 Unidad de Apoyo a la Investigacion Gerencia Asistencial Atencion Primaria, Red de investigación en Servicios de Salud en enfermedades cronicas REDISSEC, Servicio Madrileño de Salud, Calle San Martín de Porres, 6, 28035 Madrid, Spain; 5 Unidad de Apoyo Técnico, Unidad de Apoyo a la Investigacion, Gerencia Asistencial de Atención Primaria, Servicio Madrileño de Salud, Calle San Martín de Porres 6, 28035 Madrid, Spain; 6 Unidad de Apoyo a la Investigacion Gerencia Asistencial de Atencion Primaria, Red de investigacion en Servicios de Salud en enfermedades cronicas REDISSEC, Servicio Madrileño de Salud, Calle San Martín de Porres, 6, 28035 Madrid, Spain; 7 Centro de Salud los Cármenes, Servicio Madrileño de Salud, Calle Vía Carpetana, 202, 28047 Madrid, Spain; 8 Centro de Salud Espronceda, Servicio Madrileño de Salud, Calle Espronceda, 24, 28003 Madrid, Spain; 9 Centro de Salud Isabel II, Servicio Madrileño de Salud, Calle Isabel II, s/n, 28980 Parla, Spain; 10 Centro de Salud Getafe Norte, Servicio Madrileño de Salud, Calle Rigoberta Menchú, 2, 28903 Getafe, Spain; 11 Centro de Salud Las Margaritas, Servicio Madrileño de Salud, Calle Calle Magallanes, 6, 28903 Getafe, Spain; 12 Centro de Salud Los Yébenes, Servicio Madrileño de Salud, Calle los Yébenes, 46, 28047 Madrid, Spain; 13 Centro de Salud Las Margaritas, Servicio Madrileño de Salud, Calle Magallanes, 6, 28903 Getafe, Spain; 14 Centro de Salud Ciempozuelos, Servicio Madrileño de Salud, Calle Padre Benito Menni, s/n, 28350 Ciempozuelos, Spain; 15 Seccion de Prevencion y Promocion de la Salud del Servicio Territorial de Salud Pública Área 9, Servicio Madrileño de Salud, Avenida Portugal, 2, 28916 Leganés, Spain; 16 Centro de Salud Lucero, |Servicio Madrileño de Salud, Calle Latina, 14, 28047 Madrid, Spain; 17 Unidad de Apoyo a la Investigación Gerencia Asistencial Atencion Primaria, Servicio Madrileño de Salud, Calle San Martín de Porres, 6, 28035 Madrid, Spain

**Keywords:** Breast-feeding, Primary healthcare, Health education

## Abstract

**Background:**

The World Health Organization leads a global strategy to promote the initiation and maintenance of breast-feeding.

Existing literature shows that education and supportive interventions, both for breast-feeding mothers as well as for healthcare professionals, can increase the proportion of women that use exclusive breast-feeding, however, more evidence is needed on the effectiveness of group interventions.

**Methods:**

This study involves a community-based cluster randomised trial conducted at Primary Healthcare Centres in the Community of Madrid (Spain). The project aims to evaluate the effectiveness of an educational group intervention performed by primary healthcare professionals in increasing the proportion of mother-infant pairs using exclusive breastfeeding at six months compared to routine practice.

The number of patients required will be 432 (216 in each arm). All mother-infant pairs using exclusive breastfeeding that seek care or information at healthcare centres will be included, as long as the infant is not older than four weeks, and the mother has used exclusive breastfeeding in the last 24 h and who gives consent to participate.

The main response variable is mother-infant pairs using exclusive breast-feeding at six months.

Main effectiveness will be analysed by comparing the proportion of mother-infant pairs using exclusive breast-feeding at six months between the intervention group and the control group. All statistical tests will be performed with intention-to-treat. The estimation will be adjusted using an explanatory logistic regression model. A survival analysis will be used to compare the two groups using the log-rank test to assess the effect of the intervention on the duration of breastfeeding. The control of potential confounding variables will be performed through the construction of Cox regression models.

**Discussion:**

We must implement strategies with scientific evidence to improve the percentage of exclusive breast-feeding at six months in our environment as established by the WHO. Group education is an instrument used by professionals in Primary Care that favours the acquisition of skills and modification of already-acquired behaviour, all making it a potential method of choice to improve rates of exclusive breast-feeding in this period.

**Trial registration:**

The trial was registered with ClinicalTrials.gov under code number NCT01869920 (Date of registration: June 3, 2013).

## Background

### Feeding newborns: The benefits of breast-feeding

Breast-feeding is the natural way to feed newborns, and human milk is the best-adapted food for their nutritional needs. Breast-feeding (BF) is considered the most adequate way to feed newborns as it provides all of the nutrients that they need to grow and develop in optimal conditions [1]. The World Health Organization (WHO) defines three types of breast-feeding [2]: exclusive breast feeding (EBF), when the infants receive only breast milk, accepting the use of rehydration salts, drops, syrups, vitamins, minerals or medicines; predominant breast-feeding (PBF), when water or water-based liquids and/or fruit juices are added to that included in EBF; and complementary feeding (CF), when any solid or liquid foods, including formula and non-human milk, are added.

BF presents various advantages for newborns, their families and the society in general, taking into account not only the health benefits, but also those that affect social, economic and environmental areas. The short-term benefits for newborns include a better nutritional and immunological state, better intestinal function, and a better establishment of attachment and increased psychological wellbeing [3, 4]. On the long-term, it decreases the risk of diabetes mellitus, obesity, heart disease in adults, certain allergy and inflammatory diseases, and it influences neurocognitive development [5–10]. For mothers, on the short-term it favours uterine involution, weight loss, decreased risk of uterine haemorrhaging, anaemia, hypertension and post-partum depression. On the long-term it acts as a protective factor against the risk of osteoporosis and breast and ovary cancer [4].

Among the social, economic and environmental benefits, BF incurs less costs both for families as well as for health systems while it also decreases the amount of residues and consumption of energy [11].

### Recommendations regarding the promotion of breast-feeding

Efforts are currently being made, both in the health sector as well as in non-health sectors, to promote the initiation and maintenance of BF. A global strategy led by the WHO has been launched that manifests the importance of nutrition in the first months and years of life and the fundamental role that correct feeding practices play in achieving optimal health. It also highlights the need to create comprehensive national policies that guarantee that health services protect, foment and support BF and opportune and adequate complementary feeding without interruption of BF. As a global Public Health strategy, EBF is recommended during the first six months of life, with posterior introduction of complementary feeding while continuing with BF up to 2 years of age or more if the mother would like to [12, 13].

Breast-feeding is a natural act and a learned behaviour that is possible for most mothers as long as they have adequate information, support from their families, communities and healthcare systems, as well as protective public policies for BF [12]. The promotion of BF must also incorporate a cultural focus that takes into account the effect that family environments can have, especially from mothers, grandmothers and partners [12, 14].

### Prevalence of breast-feeding in developed countries: Associated factors

In spite of the broad dissemination of recommendations to promote EBF and its benefits, only 35% of newborns around the world receive EBF after the first three or four months of life [15]. The rates are much lower than those set out by the WHO in European countries, and specifically, in Spain the rates are similar to the rest of the developed countries, with 24.72% EBF at six months, and slightly lower in the Region of Madrid (23.54%) [16].

In Spain the average length of breastfeeding is around 3–5 months. The greater number of abandonments of EBF takes place during the first 4 weeks, (sometimes up to one third of cases) and between the 3rd and 4th months of life, which coincides with the imminent return of the mother to work [17–20].

According to the results of an observational study in Spain the factors that influence the continuity of EBF up to six months are diverse: lack of confidence to breast-feed, pain, suction problems, cracks, infections, ingurgitation, mastitis, baby crying, type of birth, execution of early skin to skin contact, bad experience with prior breast-feeding, comments by family members and/or friends, aspects related with going back to work, and the socio-economic and education level of the mother [21, 22]. Other authors point out the possible effect that the model of the breast-feeding mothers’ mothers, women that lived during periods of setbacks regarding the prevalence of breast-feeding, many of whom most likely did not breast-feed, could have on the breast-feeding mother’s [14].

### Strategies to foment breast-feeding

To improve breast-feeding rates, strategies directed at professionals, breast-feeding mothers and those around them are being implemented.

The WHO and UNICEF launched the BFHI (Baby Friendly Hospital Initiative) strategy in 1991 with the objective to encourage hospitals, health departments and maternity wards to adopt practices that protect, promote and support EBF from birth [23]. Since conception, the strategy has spread into other healthcare settings in different countries.

On a national level (in Spain), a BFHI Healthcare centre strategy was created, adapting the aforementioned to the reality of Primary Healthcare [24]. The National Sexual and Reproductive Health Strategy of the Ministry of Health in 2010 included the promotion of BF [25].

On a regional level, the Health Department of Madrid and UNICEF withhold an agreement of collaboration to execute and consolidate changes in clinical practice following the criteria set forth in the BFHI initiative. Breast-feeding promotion committees have been created to implement the recommended good practices, EBF culture is being promoted and primary healthcare and hospital professionals of the Region of Madrid are being trained.

In primary healthcare, within the framework established by the Standardized Service Portfolio, individual interventions have been implemented to foment BF that are similar to those executed and evaluated in other regions in Spain [26]. Also, with the Health Promotion and Prevention Plan of the General Directorate of Primary Healthcare, the objective of promoting and supporting breast-feeding is included, encouraging its use during the first six months of life [27].

Two Cochrane reviews were published in 2008 related to BF.

The review by Dyson L, McCormick F y Renfrew MJ on interventions to promote the initiation of breast-feeding included seven studies with 1388 women and its results reflected the fact that education on breast-feeding had a significant effect on the increase of the rates of initiation compared with habitual practices (RR 1.53; CI of 95%: 1.25 to 1.88) [28].

The review by Britton C et al. on support for breast-feeding included 34 controlled, randomized and quasi-randomized clinical trials that compared the additional support for mothers that breast-fed with habitual practices, on a sample of 29,385 mother-child pairs from 14 countries. The results reflected that all of the forms of additional support analysed together showed an increase in the duration of any type of breast-feeding (RR of interruption of BF before six months of 0.91; CI of 95%: 0.86 to 0.96). All of the forms of additional support together had a greater effect on the duration of EBF than on any BF type (RR 0.81; CI of 95%: 0.74 to 0.89). Professional and non-professional support together significantly prolonged the duration of any BF (RR before 4 to six weeks 0.65; CI of 95%: 0.51 to 0.82; RR before 2 months 0.74; CI of 95%: 0.66 to 0.83). EBF was significantly prolonged with training by the WHO/UNICEF (RR 0.69; CI of 95%: 0.52 to 0.91). The review also concluded that more evidence is needed on the effectiveness of group interventions [29]. Specifically, the interventions that have shown to be effective to increase EBF on the short- and long-term include any form of support provided by healthcare professionals and non-healthcare professionals, individual interventions with an education or supportive component, and combined interventions related with pregnancy and the immediate post-partum period [29–32].

In the Region of Madrid, a clinical trial in Primary Healthcare (PHC) has been conducted to evaluate a strategy of implementation of clinical practice guidelines for BF directed at professionals. The experimental intervention has achieved an improvement in the rates of EBF at six months of 16% [33].

A multidisciplinary group of experts from the General Directorate of Primary Healthcare has designed an education project for group health that uses the promotion of BF as a medium to improve mother-infant health. As concluded in the Cochrane review by Britton C, more evidence is needed on the effectiveness of group interventions.

Our research proposal is The PROLACT study, which aims to measure the effectiveness of an educational group intervention in Primary Healthcare Centres.

By evaluating the effectiveness of this type of intervention, we will be able to produce rigorous projects that guarantee attainment of the proposed objectives while also finding out the results produced in the patients.

BF is a human behaviour that is greatly influenced by that which occurs in the community to which a mother belongs, and the effect can be contaminated from one mother to another. Likewise, when a professional is trained, it is rare that they would have an attitude unlike that of the mothers. Because we consider inevitable a ‘contamination’ effect of influence on each other between professionals working in the same centre, a cluster design has been chosen so that the interventions that do or do not take place will include all the professionals in each Primary Healthcare Centre (PHCC).

## Methods/design

### Objectives

The primary objective of this study is to evaluate the effectiveness of an educational group intervention in primary healthcare, compared to habitual practice (usual care) in increasing the proportion of mother-child pairs that use EBF up to six months of age.

The secondary objectives are:

- To compare the effectiveness of an educational group intervention in primary healthcare with usual care to increase the proportion of mother-child pairs that use PBF up to six months of age.
- To describe women’s adherence and degree of satisfaction with the educational group intervention.
- To describe the reasons for abandonment of breast-feeding in both study groups. To describe the reasons of breastfeeding abandonment in both study groups.
- To explore the predictive factors of maintenance EBF at six months of age.

### Design of the study

This study is a community, multicentre, parallel clinical trial, randomised by clusters, that compares usual care and an education strategy performed by primary healthcare professionals. The intervention will be carried out with mother-infant pairs in ten PHCCs in the Community of Madrid (Spain).

The randomization units will be the PHCCs (clusters). The units of analysis are the mother-infant pairs attended to by the PHCCs. This design by clusters minimises possible contamination effects between centres.

### Subjects of the study

Mother-infant pairs using EBF, that seek care or information at any PHC consultation before the first month of life of the newborn and that give their consent to participate in the study.

#### Inclusion criteria

Professionals of the randomization unit (CS):

- Minimum of 2 years working as a professional in PHC.
- Have no intention of transferring during the study period.
- Provide healthcare to the infant population within their daily healthcare workload.
- Sign the researcher agreement.

Mother-infant dyad:

- Mothers of full-term newborns and that use EBF ≥ 18 years of age at the selection visit
- Full-term newborns (≥ 37 weeks of gestation), with a birth weight > 2.5 kg and with the newborn’s age ≤ 4 weeks.

#### Exclusion criteria

Professionals of the randomization unit (CS):

- Those that reject participation in the study.

Mother-child pairs:

- Clinical conditions of the mother that contraindicate breast-feeding: active tuberculosis, active chicken pox, active Herpes lesions on breasts, Chagas disease; human immunodeficiency virus (HIV); human T-lymphotropic virus (HTLV) I and II; substance abuse; mother in treatment with radioactive isotopes or chemotherapy or antimetabolite drugs.
- Clinical conditions of the child that complicate or impede breast-feeding, orofacial malformations.
- Clinical conditions of the child that contraindicate breast-feeding: classic galactosemia (galactose-1-phosphate uridyltransferase deficiency) in newborns.
- Infants that have been initiated on complementary feeding.
- Women that do not consent to participating in the study.
- Impossibility of the mother to come to the visits proposed for the conduction of the study.
- Difficulties with the language that impede or complicate communication between the mother and the healthcare professionals.
- Women that are participating in another research study during the study period.

### Sample size

To calculate the sample size, we believe that the educational group intervention can increase the proportion of mother/infant pairs that use EBF at six months by 15%. Assuming that 24% of mothers use EBF at six months (National Health Survey (2006). National Statistics Institute http://www.ine.es), a type I error of 5% and a strength of 80%, we need a sample of 150 mother/infant pairs in each group. As this is a randomized design with conglomerates (each conglomerate is a health centre), the sample size needs to be increased taking into account the effect of the design (ED = 1+ (ñ-1)*CCI, where ñ is the average size of each cluster and CCI is the intra-class coefficient of correlation).

Considering a CCI of 0.01 and an average size of 30 pairs per centre, the effect of the design is 1.29. Taking this data into account, the sample size increases to 194 pairs in each group. If we then take into account the loss rate of 10%, the total size surpasses 432 pairs (216 for each branch).

### Randomisation

The randomisation unit will be the healthcare centres.

An independent statistician will carry out the randomised allocation to form groups of the same size from a list of the participating healthcare centres.

**Fig. 1:**
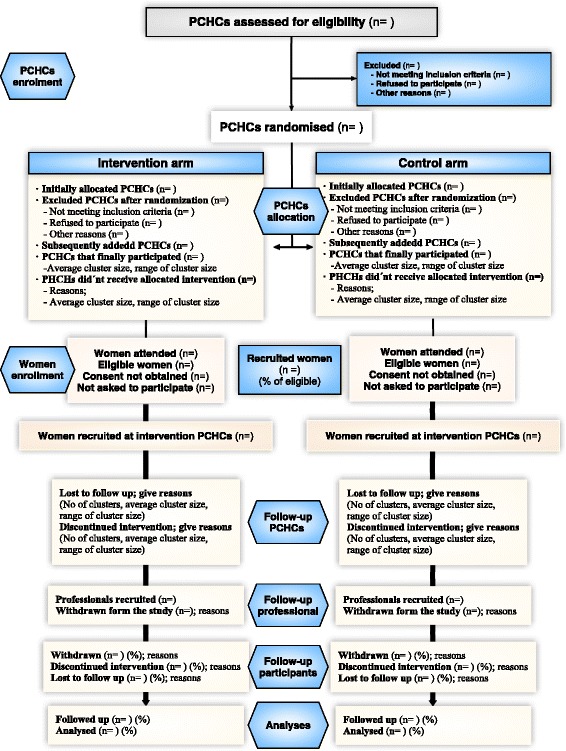
Project structure

Posteriorly, within each unit, the mother-infant pairs will be selected by consecutive sampling, until the number is reached to form the cluster. Figure 1 describes how the pairs are recruited. During the consultations, the mothers will be informed about the study and asked if they would like to participate. All mothers that would like to have to sign a consent form and it will be confirmed that they meet all inclusion/exclusion criteria.

### Masking

In a study of this type it is impossible to mask the intervention. The analysis data will be performed by independent professionals blinded to the allocation group.

### Intervention

The healthcare professionals will carry out the recruitment, they will collect the study variables, apply the assigned intervention and perform all follow-up (consultation and/or telephone). Each one of them will only carry out one type of intervention: experimental or control. To avoid the differences in the measurement of variables and the application of the intervention as much as possible, previous training will be executed.

The organization and functioning of the health services will not be altered significantly to carry out the study, as the control intervention is the habitual practice and the intervention for the treatment group will be planned and conducted just like any other educational group intervention in the core of the primary healthcare team. The Working Plan is shown in Table 1.

**Table 1:** Project Working Plan

Visit	Randomisation:2 months before visit 1	Trainig: 1–2 months before visit 1	Visit 1: < 1 month after birth	Visit 2: 2 months after birth	Visit 3: 3 months after birth	Visit 4: 4 months after birth	Visit 5: 5 months after birth	Visit 6: 6 months after birth
Study procedure***s***								
Randomisation	**X**							
Professional training		**X**						
Informed consent			**X**					
Recruitment			**X**					
Demographics			**X**					
Telephone contact				**X**	**X**	**X**	**X**	**X**
Interventions								
Standard practice				**X**		**X**		**X**
PROLACT intervention			**X**		**X**		**X**	
Outcomes measures								
EBF			**X**	**X**	**X**	**X**	**X**	**X**
PBF			**X**	**X**	**X**	**X**	**X**	**X**
CF			**X**	**X**	**X**	**X**	**X**	**X**
Reasons for abandoning breastfeeding			**X**	**X**	**X**	**X**	**X**	**X**
Acceptability of intervention			**X**					
Adherence			**X**		**X**		**X**	
Satisfaction							**X**	

The Standardized Services Portfolio for Primary Healthcare of the Community of Madrid defines four services of assistance to the supervision and development of the infant population. These services are the promotion of health habits, monitoring of development, systemic vaccinations and early detection of childhood problems.

These services are carried out transversally throughout the scheduled revisions [available at http://www.madrid.org/cs/Satellite?c=CM_Publicaciones_FA&cid=1142521116585&idConsejeria=1109266187266&idListConsj=1109265444710&idPagina=1343067104390&language=es&pagename=ComunidadMadrid%2FEstru].

#### Control group (usual care)

Usual care includes individual counselling about the benefits of maintenance exclusive breast-feeding during the first six months of the baby and about the introduction of complementary food after that. The mother should attend to the PHC at least twice, once before six months of age and another between six and 12 months of age.

#### Intervention group (educational group intervention)

This is an educational group intervention based on the workshop on breast-feeding designed by the expert group of the General Directorate of Primary Healthcare of the Madrid Health Department. It is structured in six weekly sessions of 120 min each. Its objectives are the acquisition, reinforcement and/or consolidation of the necessary knowledge and skills to initiate and maintain EBF as the newborns feeding, as well as the development of a positive attitude regarding breast-feeding. It consists of theoretical and practical content with the active participation of the mothers in the discussion group and the learning of skills through the direct practice of breast-feeding.

The activities will begin around the first month of life of the child (the maximum peak of abandonment according to existing studies) [20–22, 24] and it will take six weeks.

The mothers will be offered during the training session the possibility to come with the person that most influences their decision to breast-feed (social support) [15, 17].

Follow-up will be conducted through regular revisions according to the protocol and telephone calls to collect the study variables.

### Variables

#### Outcome variables

The main outcome variable is the percentage of infants who continue exclusively breastfed at six months.

The secondary outcome variables will be:

- Type of feeding at six months (categorical: EBF, PBF, CF).
- Duration of EBF (days) = (Date of abandonment EBF – Date of birth of infant).
- Reasons for abandonment of BF (semi-structured questionnaire created for the study).
- Number of group education sessions to which the dyad attends. Adequate adherence is considered as attendance to at least 85% of the planned sessions.
- Degree of satisfaction with the educational group intervention (measurement instrument: SERVQUAL).

The prognostic variables will be:

- For the professional: age (years), sex, breastfeeding training received.
- For the mother: age (years); education level (low – primary studies only; medium - high school completed, technical training or other non-university studies completed; high - university level education completed); income level; nationality; working situation of the mother (if she works outside the home and the number of hours per working day/ days per week); living situation with partner; obstetric history (pregnancies/abortions/live births); type of birth (vaginal/caesarean; single/multiple); Previous breastfeeding experience; peso(kg); size(cm); smoker (yes/no).
- For the newborn: single foetus (YES/NO); sex; birth-weight (grams); discharged from hospital with mother (YES/NO); APGAR test, separation from the mother during hospital stay (YES/NO); breast-fed during first hours after birth (YES/NO).
- Regarding breast-feeding: intention to breast-feed (only mother’s milk/only formula/both/I don’t know); received support for breast-feeding during pregnancy (YES/NO); received support for breast-feeding in the hospital (assessment of the attention received in the hospital regarding breast-feeding using the BFHI test); family support measured using the Apgar family test [34]; if breast-feeding took place in the first 30 min.; self-efficacy in breast-feeding measured using the Breastfeeding Self-Efficacy Scale-Short Form (BSES-SF) (test validated in Spain with a global score of 0 to 100); cracks or mastitis in the first 8 days; schedule limitation of feedings.

### Data collection method

The information will be incorporated into an electronic case report form (eCRF), ensuring confidentiality and anonymity, and guaranteeing compliance with current regulations. The age of the mothers that reject participation in the study will be recorded.

Likewise, the losses and abandonments during the study and their causes will be recorded. The mothers will be called by telephone to invite them to attend the education group sessions and to the monitoring of the control group.

Through the combination of our web-based, instantaneous electronic validation, the main researcher daily visual cross-validation of the data for complex errors, and regular on-site monitoring, the quality and completeness of the data will be reflective of the state of the art in clinical trials.

All Principal Investigators will be given access to the cleaned data sets. Project data sets will be housed on the Project Accept Web site created for the study, and all data sets will be password protected. Project Principal Investigators will have direct access to their own site’s data sets, and will have access to other sites data by request. To ensure confidentiality, data dispersed to project team members will be blinded of any identifying participant information.

### Statistical analysis

The database will be filtered before the statistical analysis is performed to improve the quality of the data collected. The use of the design by conglomerates will be taken into account in all phases of the analysis, especially when calculating the confidence intervals of the estimations and in the hypothesis tests. The following will be carried out:

1. Descriptive analysis of the demographic and baseline characteristics of the subjects of both groups. The quantitative variables will be described by their measurements of central, mean or median tendencies, in the case of asymmetrical distributions, and their measurements of dispersion, typical deviation or interquartile amplitude, respectively. The qualitative variables will be described by their proportion and confidence interval.

2. Initial comparability study of the two groups regarding their baseline characteristics, response variables and prognostic factors. The Student t test or the Mann-Whitney test will be used if the normality hypothesis is rejected for the data. If the study variables are qualitative, the Pearson’s Chi-squared test or Fisher’s exact test will be used, when applicable. In the case of inequality, the possible confounding factors will be defined by which the final analysis of the principal variable of effectiveness will be adjusted.

3. Principal analysis of effectiveness. The effect of the PROLACT intervention in the principal response variable: a comparison will be made of the proportion of mother/child pairs using EBF at six months in both groups, using Fisher’s exact test, while also estimating the confidence interval for the difference. If the confounding factors were distributed in an unequal manner in each group, a model will be constructed of explicative logistical regression where the dependent variable will be EBF at six months and the independent variable will be “treatment” group adjusted for the possible confounding factors. The analysis of effectiveness will be done by intention-to-treat.

4. Analysis of secondary effectiveness. To evaluate the effect of the educational group intervention on the duration of the different types of breast-feeding, an analysis of survival will be done, making the comparison between the two groups using the log-rank test. The control of the possible confounding variable will be done with the construction of different Cox regression models. The proportion of women that attend at least 85% of the planned sessions will be calculated to study the degree of adherence. The women’s satisfaction with the educational group intervention will be measured with a Likert-type scale.

All *p*-values below 0.05 will be considered as statistically significant for all cases.

## Discussion

Revisions of other studies show us that education and supportive interventions, both for breast-feeding women as well as for healthcare professionals, can increase the proportion of women that use EBF [29, 30, 35, 36].

As concluded in the Cochrane review by Britton C, more evidence is needed on the effectiveness of group interventions. Generalization should be preceded by the development of clinical trials that measure relevant clinical results in patients, that surpass the limitations of the studies designed to today’s date and that are conducted in the setting in which they will be implemented [29].

The majority of the interventions have been conducted in hospital settings (or maternity wards) or in countries that present socio-cultural differences [32] from Spain. Therefore, studies are needed in the contact of Primary Healthcare, with the fundamental purpose being the promotion of health lifestyle habits and where mothers-infants receive the greater part of healthcare.

Our proposal as a research group in the setting of the promotion of BF in primary healthcare is the PROLACT study, with which we aim to measure the effectiveness of educational group intervention of the General Directorate of Primary Healthcare, to later generalize its implementation in all Healthcare Centres.

The design selected is the most adequate given the characteristics of the intervention. Although a sufficient number of clusters will be selected to equilibrate the randomisation of potentially confounding factors, the following limitations must be taken into account:

- The participation of professionals in the study is voluntary and bias may be produced in the selection of the sample.

- There are healthcare centres professionals that are highly motivated regarding breast-feeding in the Community of Madrid that, during randomisation, may be assigned to the control group. Given that an intention-to-treat analysis will be performed, the mothers attended to in these centres will be analysed in the study branch in which they were assigned by randomisation, that is, the control group, regardless of the intervention that they receive in practice. This fact could generate a tendency to underestimate the effect of the intervention being studied.

- The lack of blinding in a study in which the majority of the variables are self-referred by the mothers.

- As it is a non-pharmacological intervention, there may be differences in the manner in which the different professionals carry it out. Training sessions on the educational group intervention will be carried out to minimize said differences [37].

-The observations of the result variables of the individuals from the same cluster tend to be positively correlated with those of other members of the same cluster and thus we cannot assume statistical independence [38].

## Abbreviations

BF: Breast-feeding
CF: Complementary feeding
CI: Confidence interval
EBF: Exclusive breast-feeding
eCRF: Electronic case report form
HCC: Healthcare Centre
ICC: Intra-class correlation coefficient
PBF: Predominant breast-feeding
PHC: Primary Healthcare
PHCC: Primary Healthcare Centre
RR: Relative risk
WHO: World Health Organisation

## References

[1] Proyecto de la UE sobre promoción de la lactancia en Europa. Protección, promoción y apoyo a la lactancia en Europa: plan estratégico para la acción. Comisión Europea, Dirección pública de Salud y Control de Riesgos, Luxemburgo, 2004. Disponible en: http://www.aeped.es/sites/default/files/5-europe_a_blueprint_for_action.pdf

[2] OMS (2007). Indicadores para evaluar las prácticas de alimentación del lactante y del niño pequeño: conclusiones de la reunión de consenso llevada a cabo del 6 al 8 de nov 2007 en Washington, DC. Estados Unidos.

[3] Oddy WH (2002). The impact of breastmilk on infant and child health. Breastfeed Rev.

[4] American College of Obstetricians and Gynecologists. Breastlfeeding: maternal and infant aspects. ACOG educational. Bulletin Julio. 2000:258.

[5] Harten T, Bergmann R, Kallischining G, Plagemann A (2005). Duration of Breastlfeeding and risk of overweight: a meta-analysis. Am J Epidemiol.

[6] Rudnicka AR, Owen CG, Strachan DP (2007). The effect of breastfeeding on cardiorespiratory risk factors in adult life. Pediatrics.

[7] Gdalevich M, Mimouni D, Mimouni M (2001). Breast-feeding and the risk of bronchial asthma in childhood: a systematic review with metanalysis of prospective studies. J Pediatr.

[8] Owen CG, Martin RM, Whincup PH, Smith GD, Cook DG (2006). Does breasfeeding influence risk of type 2 diabetes in later life? A quantitative analysis of published evidence. Am J Clin Nutr.

[9] Koletzko S, Sherman P, Corey M, Griffiths A, Smith C (1989). Role of infant feeding practices in developmental of Crohn’s disease in childhood. MNJ.

[10] Der G, Batty GD, Deary IJ (2006). Effect of breastfeeding on intelligence in children: prospective study, sibling pairs analysis, and meta-analysis. BMJ.

[11] “Alimentación de los lactantes y de los niños pequeños” Normas recomendadas para la UE 2005/2006). Cattaneo A, Fallon M, Kewitz G, Mikiel- Kostyra K, Robertson A. Alimentación de los lactantes y de los niños pequeños: Normas recomendadas por la Unión Europea EUNUTNET (Red Europea para la Nutrición Pública Saludable: Trabajo en red, monitorización, intervención y formación). Comisión Europea. 2005–2006.

[12] OMS (2003). Estrategia Mundial para la Alimentación del Lactante y del Niño Pequeño.

[13] US Preventive Services Task Force (2008). Primary care interventions to promote breastfeeding: U.S. preventive services task force recommendation statement. Ann Intern Med.

[14] Criado Rodriguez E, Esquilas Martín MC, San Román Muñoz P (1998). Lactancia Materna, retos y condicionantes socioculturales. Estrategia educativa. Rev Rol Enf.

[15] World Health Organization (2001). The optimal duration of exclusive breastfeeding.

[16] Ministerio de Sanidad y Política Social. Encuesta Nacional de Salud de España 2006. Disponible en: https://www.msssi.gob.es/estadEstudios/estadisticas/encuestaNacional/encuesta2006.htm.

[17] González M, Toledano J (2007). La Lactancia materna en nuestro medio: análisis de la situación. Acta Pediatr Esp.

[18] Hernandez MT. Epidemiología de la lactancia materna. Prevalencia y tendencias de la lactancia materna en el mundo y en España. En: Lactancia Materna: guía para profesionales. Comité de Lactancia Materna de la AEP. Monografías de la AEP n°5. Madrid: Ergón, 2004, p. 31–44.

[19] Hernández MT, Aguayo J (2005). La lactancia materna. Cómo promover y apoyar la lactancia materna en la práctica pediátrica. Recomendaciones del Comité de la Lactancia AEP. An Pediatr (Barc).

[20] Labarere J, Gelbert-Baudeno N, Ayral A-S (2005). Efficacy of breastfeeding support provided by trained clinicians during an early, routine, preventive visit: a prospective randomized, open trial of 226 mother- infant pairs. Pediatrics.

[21] Sacristán Martín AM, Lozano Alonso JE, Gil Costa M, Vega Alonso AT (2011). Red Centinela Sanitaria de Castilla y León. Situación actual y factores que condicionan la lactancia materna en Castilla y León Rev Pediatr Aten Primaria.

[22] Estévez González MD, Martell Cebrián D, Santana Medina R, García Villanueva E, Saavedra SP (2002). Factores relacionados con el abandono de la lactancia materna. An Esp Pediatr.

[23] World Health Organization (2009). Baby-friendly hospital initiative: revised, updated and expanded for integrated care.

[24] Grupo de Trabajo CS-IHAN. Centros de Salud IHAN (Iniciativa de Humanización de la Atención al Nacimiento y la Lactancia). Una garantía de calidad. Rev Pediatr Aten Primaria. 2009;11:513–29. Available at: http://scielo.isciii.es/pdf/pap/v11n43/12_colaboraciones.pdf

[25] Ministerio de Sanidad, Política Social e Igualdad. Estrategia Nacional de Salud Sexual y Reproductiva. Madrid: Ministerio de Sanidad, Política Social e Igualdad; 2011. (Consulted on 23/10/2014). Available at: http://www.msssi.gob.es/organizacion/sns/planCalidadSNS/pdf/equidad/ENSSR.pdf].

[26] Rodríguez Martínez G, Fuertes Fernández-Espinar J, Samper Villagrasa MP, Broto Cosculluela P, Collado Hernández MP, Sebastián Bonel MF (2008). Programas de intervención para promocionar la lactancia materna. Proyecto PALMA Acta Pediatr Esp.

[27] Servicio Madrileño de Salud (2011). Dirección General de Atención Primaria. Plan de Promoción de la Salud y Prevención.

[28] Dyson L, McCormick F, Renfrew MJ. Intervenciones para promover el inicio de la lactancia materna (Revisión Cochrane traducida). En: La Biblioteca Cochrane Plus, 2008 Número 2. Oxford: Update Software Ltd. Disponible en: http://onlinelibrary.wiley.com/doi/10.1002/14651858.CD001688.pub2/abstract.

[29] Britton C, McCormick FM, Renfrew MJ, Wade A, King SE (2007). Apoyo para la lactancia maternal. Cochrane database of systematic reviews.

[30] Imdad A, Yakoob MY, Bhutta ZA (2011). Effect of breasteeding promotion interventions on breastfeeding rates, with special focus on developing countries. BMC Public Health.

[31] Chung M, Raman G, Trikalinos T, Lau T, Ip S (2008). Interventions in primary care to promote breastfeeding: an evidence review for the U.S. preventive services task force. Ann Intern Med.

[32] Hannula L, Kaunonen M, Tarkka MT (2008). A systematic review of professional support interventions for breastfeeding. J Clin Nurs.

[33] Martín-Iglesias S, Del-Cura-González I, Sanz-Cuesta T, Arana-Cañedo Argüelles C, Rumayor-Zarzuelo M, Álvarez-de la Riva M (2011). Effectiveness of an implementation strategy for a breastfeeding guideline in primary care: cluster randomised trial. BMC Fam Pract.

[34] Bellón Saameño JA, Delgado Sánchez A (1996). Luna del Castillo JD. Validez y fiabilidad del cuestionario de función familiar. Apgar familiar. Aten Primaria.

[35] Palomares Gimeno MJ, Fabregat Ferrer E, Folch Manuel S, Escrig García B, Escoín Peña F, Gil SC (2011). Apoyo a la lactancia materna en una zona básica de salud; prevalencia y factores sociosanitarios relacionados. Rev Pediatr Aten Primaria.

[36] Spiby H, McCormick F, Wallace L, Renfrew MJ, D'Souza L, Dyson L (2009). A systematic review of education and evidence-based practice interventions with Elath professionals and breast feeding counsellors on duration of breast feeding. Midwifery.

[37] Boutron I (2008). Extending the CONSORT statement to randomized trials of nonpharmacologic treatment: explanation and elaboration. Ann Intern Med.

[38] Adams G, Gulliford MC, Ukoumunne OC, Eldridge S, Chinn S, Campbell MJ (2004). Patterns of intracluster correlation from primary care research to inform study design and analysis. J Clin Epidemiol.

